# Spatial analysis of factors associated with HIV infection among young people in Uganda, 2011

**DOI:** 10.1186/1471-2458-14-555

**Published:** 2014-06-05

**Authors:** Lucy A Chimoyi, Eustasius Musenge

**Affiliations:** 1 Division of Epidemiology and Biostatistics, School of Public Health, Faculty of Health Sciences, University of the Witwatersrand, Johannesburg, South Africa

**Keywords:** HIV/AIDS, Spatial, Clusters, Uganda, Young people, 15-24 years

## Abstract

**Background:**

The HIV epidemic in East Africa is of public health importance with an increasing number of young people getting infected. This study sought to identify spatial clusters and examine the geographical variation of HIV infection at a regional level while accounting for risk factors associated with HIV/AIDS among young people in Uganda.

**Methods:**

A secondary data analysis was conducted on a survey cross-sectional design whose data were obtained from the 2011 Uganda Demographic and Health Survey (DHS) and AIDS Indicator Survey (AIS) for 7 518 young people aged 15-24 years. The analysis was performed in three stages while incorporating population survey sampling weights. Maximum likelihood-based logistic regression models were used to explore the non-spatially adjusted factors associated with HIV infection. Spatial scan statistic was used to identify geographical clusters of elevated HIV infections which justified modelling using a spatial random effects model by Bayesian-based logistic regression models.

**Results:**

In this study, 309/533 HIV sero-positive female participants were selected with majority residing in the rural areas [386(72%)]. Compared to singles, those currently [Adjusted Odds Ratio (AOR) =3.64; (95% CI; 1.25-10.27)] and previously married [AOR = 5.62; (95% CI: 1.52-20.75)] participants had significantly higher likelihood of HIV infections. Sexually Transmitted Infections [AOR = 2.21; (95% CI: 1.35-3.60)] were more than twice likely associated with HIV infection. One significant (p < 0.05) primary cluster of HIV prevalence around central Uganda emerged from the SaTScan cluster analysis. Spatial analysis disclosed behavioural factors associated with greater odds of HIV infection such as; alcohol use before sexual intercourse [Posterior Odds Ratio (POR) =1.32; 95% (BCI: 1.11-1.63)]. Condom use [POR = 0.54; (95% BCI: 0.41-0.69)] and circumcision [POR = 0.66; (95% BCI: 0.45-0.99)] provided a protective effect against HIV.

**Conclusions:**

The study revealed associations between high-risk sexual behaviour and HIV infection. Behavioural change interventions should therefore be pertinent to the prevention of HIV. Spatial analysis further revealed a significant HIV cluster towards the Central and Eastern areas of Uganda. We propose that interventions targeting young people should initially focus on these regions and subsequently spread out across Uganda.

## Background

HIV/AIDS remains a serious public health concern among the youth aged 15-24 years in Sub-Saharan Africa (SSA) where the epidemiology varies across regions [1,2]. An estimated 50% of all new infections occur in this age-group [3]. In East Africa, the HIV-prevalence in adults ranges from 3-7% according to the 2010 Joint United Nations Program on HIV/AIDS with females disproportionately affected than males [4,5]. The adult HIV prevalence in Uganda is the highest in East Africa but has steadily declined from 18% to 6.1% and has stabilized for a period of ten years [6,7]. The AIDS Indicator Survey (AIS) reported a HIV prevalence of 3.7% for young persons aged 15-24 years [8]. A recent study in Uganda has however shown increased HIV prevalence as a result of increased high-risk sexual behaviour [7]. Various behavioural factors have been forwarded as observations for this increase in HIV prevalence in Uganda and these include: early coital debut, multiple and concurrent sexual partnerships, lack of condom use and alcohol consumption [9-14]. Similarly, biological factors including presence of an STI and low male circumcision rates were reportedly associated with HIV/AIDS [4,15].

Studies have demonstrated that different HIV risk factors associated with young people may enable researchers explain their varied HIV prevalence [16,17]. A study conducted in Kisumu, Kenya investigating the spatial distribution of STI and sexual behaviours in 18-24 year-old sexually active men using cluster analysis revealed several high and low rate geographical clusters of HIV with one significant cluster of men who used condoms less frequently [2]. In Durban, South Africa a study investigating the geographical variations of three STIs among a cohort of sexually active women using SaTScan revealed that STI incidence and prevalence was clustered in localized locations which overlapped with areas of high HIV prevalence [18]. Spatial variations of HIV infections among women in KwaZulu Natal province, South Africa, were investigated by use of geo-additive models. These identified significant spatial patterns that could not be accounted for by standard regression procedures [19]. Kulldorff spatial scan has also been used to investigate small geographical patterns and HIV clustering in a rural South African population revealing geographical variation and significant clusters of HIV prevalence in a fairly homogenous population [20]. In Nigeria, Exploratory Spatial Data Analytical (ESDA) techniques were used to determine variation of HIV/AIDS by revealing significant clusters of localized HIV/AIDS [21]. Spatial cluster techniques used to identify clusters of HIV infection enhanced the understanding of the determinants of HIV infection and geographic patterns which contributed to improved allocation of public resources in the Democratic Republic of Congo [22].

However, limited spatial research has been conducted in Uganda using geographical analysis to better understand the spatial epidemiology of HIV/AIDS and therefore provide health officials with guidance on formulating appropriate interventions in young people [23]. It is important to note that HIV/AIDS has a geographical structure that determines its epidemiology, a characteristic of spatially correlated data [19,24]. Spatially correlated outcomes have common exposures that influence transmission in neighbouring locations [25]. This usually creates spatial heterogeneity of diseases on a community, regional or national level [23,26]. Spatial analysis therefore takes into account these variations providing parameter estimates and predictions [27,28] that can be used to produce spatial risk maps with the outcomes of interest in areas otherwise not sampled [29]. Therefore, the use of spatial rather than standard regression models is suitable for accounting for these variations at regional level in Uganda. Understanding the contribution of geographical analysis on HIV prevalence particularly in young people is important. A number of articles have demonstrated the various risk factors associated with HIV prevalence among young people in Uganda [6,7,30,31]. This paper used data from population-based sample surveys to explore the effects of socio-demographic and behavioural characteristics on HIV prevalence among young people. In addition, the study employed spatial analysis to determine factors associated with HIV/AIDS among young people in Uganda. Cluster techniques identified high and low-risk areas of HIV infection in Uganda. In addition spatial regression modelling was used to compare risk factors associated with HIV infection before and after adjusting for geographical differences.

## Methods

### Study design, sampling design and data collection

This cross-sectional study utilized secondary data from the 2011 Uganda Demographic and Health Survey (UDHS) and AIDS Indicator Survey (AIS) where data was extracted for participants aged 15 to 24 years. In the primary study, respondents were selected using a two-stage sampling process from stratified urban and rural areas. The first stage involved the selection of a number of Enumeration Areas (EAs) selected from a list of DHS clusters created from a recent population census [32,33]. The second stage involved selecting households from a household list in the selected EAs where all household members of reproductive age 15-49 (females) and 15-54 (males) were selected [32,33].

Ethical clearance for this study was obtained from the University of the Witwatersrand Ethics Committee on Human Subjects (M120856). Permission was also sought from the MEASURE DHS ICF International to use the data for secondary analysis.

### HIV data

HIV data was obtained from the UAIS conducted in 2011. Blood collection through finger pricking or venous flow for HIV testing was provided voluntarily by individuals whose households were selected during the survey. Home-based rapid tests and dried blood spots were used for individuals who consented to venous flow and those who preferred finger- pricking respectively. Subsequent testing was performed using Murex and Vironostika Uniform II O to confirm sero-status and ANILAB to resolve discordant results [8].

### Selection of sample

The sample used in this study was acquired through the merging and appending of five datasets downloaded from the MEASURE DHS website. These were; the standard individual dataset containing socio-demographic information for males and females, a household member dataset with information on all household members, AIS dataset which had indicators for effective monitoring of HIV, and geo-referenced information on the households that participated in the survey. A final dataset which included respondents who were between 15-24 years of age having a GPS co-ordinate and HIV result was used in the final analysis.

### Measurement of variables

The dependent variable was HIV sero-status categorized as being HIV positive/negative. The independent variables examined were socio-demographic, biological and behavioural. Socio-demographic variables were gender (male/female), place of residence (urban/rural), education level (no education/primary/secondary/higher), religion (Christian/Muslim/Traditional/None), marital status (Never married/married/separated/divorced/widowed) and Circumcision (yes/no). Behavioural factors included alcohol consumption in the past 12 months (yes/no), multiple sexual partners defined as having more than one sexual partner in the past 12 months (yes/no), condom use in the past one year (yes/no), transactional sex defined as engaging in sex in exchange for goods and money (yes/no) and coital debut (none/8-10/11-14/15-19/20-24). Young coital debut was defined as age at first sexual encounter below 20 years of age. Biological factors which included the presence of a Sexually Transmitted Infection (STI) or its symptom in the past 12 months (yes/no) were examined. Social Economic Status (SES) was assessed using the wealth quintile. Lowest, second/middle/fourth and highest was categorized as low, middle and high SES respectively. Media exposure looked at respondents who were not, irregularly and regularly exposed to radio, television or newspapers respectively.

### Data analysis

Data was analysed using STATA 12 [34] and BayesX [35] software for non-spatial and spatial analysis respectively. Population size adjusting sampling weights at cluster level were also included during the analysis. Descriptive measures were used to summarize the overall characteristics of the study participants in the study area using frequencies and percentages for categorical variables and median (interquartile range) for continuous variables. Likewise, proportions of HIV sero-positive were also reported after adjusting for sampling weights. The statistical significance of apparent associations between potential risk factors and HIV prevalence was explored using chi-square and independent *t*-test for categorical and continuous variables respectively. Unadjusted and adjusted multiple logistic regression analyses were used to determine associations between the outcome and risk factors. Significant associations (p-value <0.05) from the univariate analysis were included into the final multivariable model. A multiple variable logistic regression analysis was performed without a spatial component to determine factors associated with HIV prevalence as well as adjusting for any potential confounding. This analysis was restricted to participants whose cluster centroid coordinates were collected during the survey and also consented to HIV testing and received the results.

### Spatial scan statistic

To identify and detect clusters of HIV in the study area, a Poisson-based spatial scan statistic was employed to adjust for the underlying populations in each survey cluster using the spatial scan statistic function of the SaTScan™ software version 9.0 [27]. The spatial scan statistic identified these geographical clusters by taking into consideration the rates of nearby clusters across multiple spatial scales, minimizing the potential for error resulting from the small sample sizes within each individual cluster of households [22]. The Kulldorff's SaTScan program has been widely used in public health research for applying a simple statistic in identifying spatial clusters based on geographic coordinates [36]. The spatial scan method uses a circular window which moves across the map and at each position; the radius of the circular window varies repeatedly from zero up to a set maximum radius of 50 which restricts the maximum size of the window from exceeding 50% of the total study population [37]. In this study, high rate where the observed cases exceeded the expected cases and low rate clusters where the expected cases exceeded observed cases were scanned [36,37]. The null hypothesis of no clusters was rejected when the p-value was less than or equal to 0.05.

### Bayesian modelling

Spatial binomial logistic regression was undertaken via Bayesian estimation based Markov chain Monte Carlo (MCMC) simulation using the BayesX software version 2.1 [35] to adjust for non-spatial and spatial random effects in the model. The spatial random effect model accounts for heterogeneity across spatial units that occur in geo-referenced data [35]. Significant risk factors from the non-spatial model were included as fixed effects for analysis in conjunction with the spatial components in the Bayesian modelling. Posterior Odds Ratios (POR) and their 95% Bayesian credible intervals (lower bound of 2.5% and 97.5%) as well as the spatial (structured) and non-spatial (unstructured) random effect models were reported. POR estimation was achieved by taking into account the autocorrelation in the structure of the data and the regional ambiguity of HIV infection in Uganda [28,29]. All statistical tests were performed using two-sided tests at the 0.05 level of significance.

### Mapping

**Figure 1:**
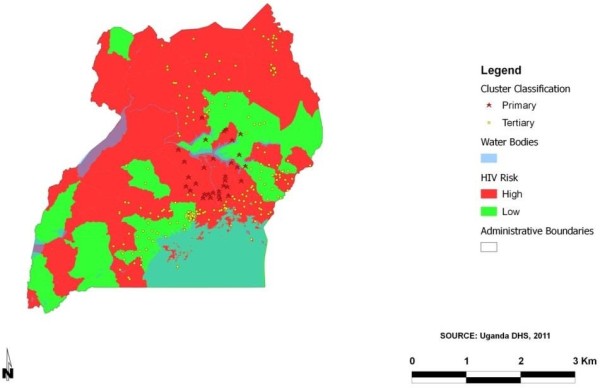
Clusters of HIV infection among study population in Uganda.

**Figure 2:**
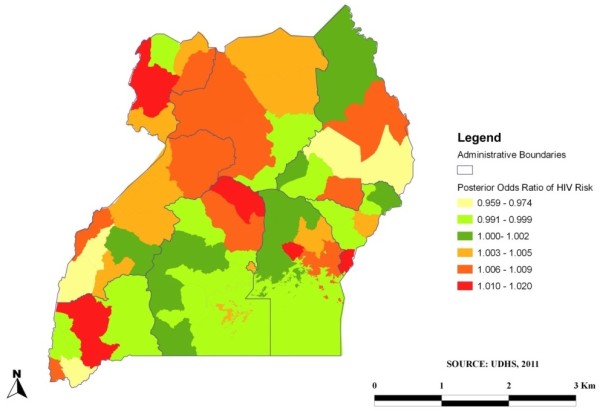
Posterior odds ratio of HIV risk among young people in Uganda.

The results from the Bayesian and cluster analysis were superimposed to produce a map that displayed low and high rate geographical clusters of HIV in the study area. High (red) and low (green) HIV risk areas were identified as shown in Figure 1. Figure 2 depicted the variations of HIV in different regions in Uganda from the BayesX output. A standard Geographical Information System (GIS) programme [38], Quantum GIS was used to translate the outputs into maps that depicted the distribution of HIV prevalence in Uganda.

## Results

A total of 7 518 participants aged 15-24 years were selected of whom 533 (7%) were HIV-positive. After adjusting for sampling weights, a proportion of 4% was HIV positive.

### Socio-demographic characteristics among HIV-positive participants

Table 1 provides a summary of the socio-demographic and behavioural characteristics of the HIV-positive participants in the study population. The mean (sd) age of this study participants was approximately 19 (2.86) years. Slightly more than half of the study participants were female (n = 309, 58%). Majority of the participants resided in the rural areas (n = 386, 72%). Compared to urban areas, the proportion of HIV positive participants was higher in the rural areas (5%) although this difference was not significant. Slightly more than half (57%) were of low socio-economic status and had an irregular exposure to media sources (n = 328, 74%). Basic literacy levels were shown by a large number of participants possessing primary school (n = 314, 59%) education and fewer individuals progressing beyond secondary school (n = 22, 6%).

**Table 1:** Descriptive characteristics of study participants

**Variable**	n(%) median(IQR)*	**Proportion of HIV+**	**P-value**^**a**^
**Age**	19(19.11-19.30)*		0.35
**Gender**			
Male	224(42)	3%	Ref
Female	309(58)	4%	0.75
**Place of residence**			
Urban	147(28)	2%	Ref
Rural	386(72)	5%	0.37
**Education level**			
None	82(15)	1%	Ref
Primary	314(59)	4%	0.21
Secondary	114(21)	2%	0.09
Tertiary	22(6)	0.2%	<0.01
**Religion**			
None	3(2)	<0.01%	Ref
Catholic	218(41)	3%	0.35
Muslim	60(11)	1%	0.21
Protestant	247(46)	3%	0.33
**Marital status**			
Never Married	31(6)	0.4%	Ref
Married	319(60)	4%	0.01
Widowed	115(22)	2%	<0.01
Divorced/separated	67(12)	0.9%	<0.01
**Socio-economic status**			
High	8(2)	<0.01%	Ref
Middle	209(41)	3%	0.25
Low	288(57)	4%	0.43
**Media exposure**			
None	67(15)	0.9%	Ref
Irregular	328(74)	5%	0.98
Regular	50(11)	0.7%	0.81
**Circumcision**			
No	40(22)	1%	Ref
Yes	145(78)	4%	0.05
**Age at sex debut**	16(16.06-16.38)*		0.17
**Age at sex debut**			
None	2(0)	<0.01%	Ref
8-10	1(0)	<0.01%	0.54
11-14	80(16)	0.1%	<0.00
15-19	362(71)	5%	<0.00
20-24	68(13)	1%	<0.00
**Multiple sexual partnerships**			
No	152(29)	2%	Ref
Yes	375(71)	5%	<0.01
**Transactional sex**			
No	169(10)	4%	Ref
Yes	151(90)	0.5%	0.02
**Condom use**			
No	71(19)	1%	Ref
Yes	309(81)	5%	<0.01
**Presence of STI and symptoms**			
No	232(46)	3%	Ref
Yes	268(54)	4%	<0.01
**Alcohol use**			
No	122(32)	2%	Ref
Yes	258(68)	4%	0.01

*Median (interquartile range).

^a^P-value for bivariate associations between outcome and risk factors (chi-square/two-sample *t*-test).

### Behavioural and biological characteristics among HIV-positive participants

Majority of the participants were married (n = 319, 60%) and reported a mean coital debut age of 16 years. Circumcision rate among the young male HIV positive participants was high (n = 145, 78%). Multiple sexual partnerships among HIV positive participants (n = 371, 71%) were rife in the study population and majority of the participants used condoms during sexual encounters (n = 307, 81%). Slightly above half of the participants reported the presence of an STI or its symptoms (n = 268, 54%). 90% of the HIV positive participants reported having engaged in transactional sex. Alcohol use was rampant in this study population as more than half of the study participants reported being inebriated during sexual intercourse (n = 258, 68%).

### Identification of HIV spatial clusters in Uganda

The spatial cluster analysis detected one significant primary (Table 2) and fifteen tertiary clusters. The most likely cluster comprised of thirty two locations situated around the Central and Eastern regions of Uganda. In this area, 70 cases were detected during the study period, while the number of expected cases was 37. An Odds Ratio [OR] (2.14, 95% CI 1.63–2.81) was also estimated which implied that locations in the primary cluster were 2.14 times more likely to be HIV infected than those outside. This geographical cluster with a radius of 74.11 km covered a great part of central Uganda. The tertiary clusters were located towards the north-east, east and south-east of Uganda, as shown in Figure 1.

**Table 2:** Spatial clusters of HIV infection among young people, Uganda, 2011

**Cluster**	**Locations**	**Observed cases**	**Expected cases**	**Odds ratio**	**Likelihood ratio**	**p-value**
1(Primary)	32	70	37.21	2.14	13.52	<0.001

### Factors associated with HIV prevalence from the non-spatial binomial regression model

This section presents the results from the univariate and multivariable analysis of the factors predisposing participants to HIV infection in Uganda. The multivariable analysis presents only the variables that were significantly associated with HIV infection in the bivariate analysis in Table 3.

**Table 3:** Marginal odds ratio of factors associated with HIV infection among young people, Uganda 2011

**Variable**	**Marginal OR (95% CI) (Unadjusted)**	**P-value**	**Marginal AOR (95% CI) (Adjusted)**	**P-value**
**Education level**				
None	1.00(Reference)	-	-	
Primary	0.79(0.54-1.14)	0.21	-	
Secondary	0.68(0.44-1.07)	0.09	-	
Tertiary	0.34(0.17-0.68)	0.02	-	
**Marital status**				
Never married	1.00(Ref)	-	1.00(Ref)	-
Married	2.22(1.27-3.89)	0.01	3.64(1.23-10.72)	0.02
Previously married	6.18(3.32-11.49)	<0.01	5.62(1.52-20.73)	0.01
**Circumcision**				
No	1.00(Ref)	-	1.00(Ref)	
Yes	0.58(0.34-1.01)	0.05	0.29(0.18-0.61)	0.02
**Transactional sex**				
No	1.00(Ref)	1.00(Ref)	-	
Yes	4.14(1.31-13.13)	0.02	-	-
**Multiple Sexual partnerships**				
No	1.00(Ref)	-	1.00(Ref)	
Yes	1.94(1.42-2.65)	<0.01	1.78(0.14-13.74)	
**Condom use**				
No	1.00(Ref)	-	1.00(Ref)	
Yes	0.44(0.29-0.68)	<0.01	0.26(0.13-0.46)	0.02
**Presence of STI and symptoms**				
No	1.00(Ref)	-	1.00(Ref)	
Yes	2.62(1.97-3.49)	<0.01	2.36(1.01-4.07)	<0.01
**Alcohol use**				
No	1.00(Ref)	-	1.00(Ref)	
Yes	1.64(1.16-2.31)	0.01	1.58(1.21-2.53)	0.02
**Age at sex debut**				
None	1.00(Ref)	-	-	
8-10	2.17(0.19-25.47)	0.54	-	
11-14	17.70(4.01-78.01)	<0.01	-	
15-19	14.49(3.41-61.62)	<0.01	-	
20-24	12.17(2.72-54.55)	<0.01	-	

Young people who were previously [OR = 5.62; 95% CI (1.52-20.73), *p* = <0.01] and currently married [OR = 3.64; 95% CI (1.23-10.72), *p* = 0.01] had a higher risk of HIV infection relative to those who were never married. Higher education (tertiary) was protective against HIV infection by decreasing the likelihood significantly by 68% [OR = 0.34; 95% CI (0.17-0.68), *p* = 0.02]. As expected, circumcision among the males reduced the chances of acquiring HIV infection by 34% compared to uncircumcised males [AOR = 0.66; 95% CI (0.44-0.99), *p* = 0.02]. Risky sexual behaviour was associated with HIV infection before controlling for demographic factors. A young coital debut (below 14 years) increased the risk of HIV among young people by 58% and this risk was seen to decrease as the age at sex debut increased. Having more than one sexual partner significantly increased the risk of HIV infection [OR = 1.94; 95% CI (1.42-2.65), *p* = <0.01]. Transactional sex was positively associated with increased risk of HIV infection [OR = 4.14; 95% CI (1.31-13.13), *p* = 0.02]. Participants who reported engaging in sex while inebriated increased their chances of acquiring HIV infection by 69% when compared with those who did not use alcohol [AOR = 1.69; 95% CI (1.57-2.45), *p* = 0.02]. STIs are a risk factor for HIV infection. The results from this study indicate that young persons aged 15–24 years, who reported having a sexually transmitted infection in the past 12 months, were approximately twice as likely associated with HIV infection compared with their counterparts who did not report a sexually transmitted infection during that same period of time [AOR = 2.21; 95% CI (1.35-3.60), *p* = <0.01]. Condom use is critical for the prevention of HIV and AIDS and other sexually transmitted infections and provided 72% protection against HIV infection relative to non-users [AOR = 0.28; 95% CI (0.15-0.55), *p* = <0.01].

### Factors associated with HIV prevalence from the spatial binomial regression model

This section presents results from the multivariable analysis of the factors associated with HIV infection in Uganda after controlling for the random and spatial effects respectively. The multivariable analysis presents only the variables that were significantly associated with HIV infection in the univariate analysis (Table 4).

**Table 4:** Posterior odds ratio of factors associated with HIV infection among young people, Uganda 2011

**Variable**	**Random effect POR (95% BCI)**	**Spatial effect POR (95% BCI)**
**Marital status**		
Never married	1.00(Ref)	1.00(Ref)
Married	0.40(0.24-0.73)***	0.39(0.18-0.72)***
Divorced/separated	2.66(1.14-6.00)***	3.23(1.23-8.44)***
**Circumcision**		
No	1.00(Ref)	1.00(Ref)
Yes	0.29(0.18-0.61)***	0.64(0.48-0.84)***
**Condom use**		
No	1.00(Ref)	1.00(Ref)
Yes	0.26(0.13-0.46)***	0.54(0.41-0.69)***
**Presence of STI and symptoms**		
No	1.00(Ref)	1.00(Ref)
Yes	2.36(1.01-4.07)***	1.72(1.49-1.95)***
**Alcohol use**		
No	1.00(Ref)	1.00(Ref)
Yes	1.58(1.21-2.53)***	1.32(1.11-1.63)***
Non-spatial variance	16.34 (13.82-18.57)	
Spatial variance		15.68(11.79-18.96)

***Significant (p-values < 0.05).

Previously married participants [POR = 3.23; 95% BCI (1.23-8.44), *p* = <0.01] had higher risks of HIV infection relative to those who were never married. Adjusting for random and spatial effects provided a protective effect against HIV infection in married participants reducing the chance of HIV infection by approximately 61% [POR = 0.39; 95% BCI (0.18-0.72), *p* = <0.01]. Circumcision among males reduced HIV infection by 36% [POR = 0.64; 95% BCI (0.48-0.84), *p* = <0.01]. Risky sexual behaviour after controlling for spatial random effects was associated with HIV infection among the young people in Uganda as well. Participants who reported engaging in sex while inebriated increased their chances of acquiring HIV infection by 32% when compared with those who did not use alcohol [POR = 1.32; 95% BCI (1.11-1.63), *p* = 0.02]. Participants who reported presence of an STI in the past 12 months, had an increased chance of HIV infection by 72% compared to those with no symptoms [POR = 1.72; 95% BCI (1.49-1.95), *p* = <0.01]. Condom use significantly reduced the chance of HIV infection by 46% [POR = 0.54; 95% BCI (0.41-0.69), *p* = <0.01]. Mapping was subsequently done based on the posterior estimates from the multivariable binomial regression spatial model as shown in Figures 1 and 2. Figure 1 illustrates the areas perceived as high (red) and low (green) areas for HIV infection among young people in Uganda. This was achieved by overlaying the results from the cluster and Bayesian analysis. As expected, majority of the areas in Uganda are seen as having a high HIV prevalence. The map in Figure 2 shows the estimated posterior regional odds of HIV infection after adjusting for geographical locations. Spatial analysis revealed the regions depicted in orange and red colours with a significantly high HIV prevalence while yellow-coloured regions showed significantly lower HIV prevalence with odd ratios less than 1.

## Discussion

The aim of this study was to identify risk factors associated with HIV infection among young people aged 15-24 years, which included individual and sexual behavioural factors in relation to the individual’s geographic locality. It was found that the association between the some demographic and behavioural variables was significant in the univariate analysis but non-significant in the multivariable analysis. It is of interest to note that the effect of behavioural variables on the risk of HIV infection in the multivariable logistic models is reduced after controlling for the demographic factors. Efforts to control the spread of HIV/AIDS among young persons should focus on eradicating behavioural factors which have been known to propagate the epidemic [39].

A Bayesian framework was applied that allowed for estimating association at individual and cluster level in an integrated framework [23]. Spatial effects, calculated through this framework, greatly influenced the distribution of HIV/AIDS infection in Uganda and signified underlying factors that may not necessarily be captured by data collection tools in many surveys but are specific to certain locations which may increase or decrease the association with HIV/AIDS [40]. Therefore spatial analysis is pertinent to the understanding of disease variations in different locations.

This study supports the known perceptions that HIV/AIDS continues to be a significant public health issue in Sub-Saharan Africa and is largely propagated by high-risk sexual behaviour [4,9,11,41-43]. The estimation of HIV prevalence among young people from this study is similar to that reported in the AIS in Uganda [8] . The overall results showed that HIV risk was higher among currently and previously married individuals, low circumcision rates and high-risk sexual behaviours. These findings are consistent with those observed in previous studies based on DHS data [12,14,17,39,41,44]. A possible explanation for increased likelihood for HIV infections among divorced/separated individuals could be that previously married individuals tend to have more sexual partners than single or married individuals [45]. In addition, HIV/AIDS and associated unsafe sexual practices might contribute to a marriage breaking down, contributing to the observed association [33]. A study by Clark et al (2006) in Africa and Latin America also observed that married young persons aged 15–24 years had a higher risk of HIV infection, approximately five times when compared with their sexually active unmarried peers [46]. These young persons could most probably have married young and transitioned from virginity to frequent unprotected sex, which they would likely continue to engage in after the end of marriage. A possibility that some of the young persons could have been infected while married and could have been separated or divorced at the recognition that they were infected with HIV could also arise [39,46]. Programmes and interventions for the control of HIV/AIDS should also focus on young widowed and divorced young persons as well as promoting approriate prevention strategies such as condom use and abstinence from sexual activities in order to prevent contracting HIV or other STIs [31,39,41,46].

The results also confirmed the significant association between early sexual debut and increased likelihood of being infected with HIV. Young persons who engage in early sexual relationships are likely to have more sexual partners than their counterparts whose sexual debut occurs later predisposing them to contracting HIV and other STIs. This observation is consistent with findings from Zimbabwe which observed that a younger coital debut was associated with an increased likelihood in HIV infection compared to a later coital debut [39].

The results further confirmed that male circumcision and condom use reduced the risk of HIV infection in the study population. These findings are supported by three randomized clinical trials conducted in Kenya [15], Uganda [30] and South Africa [47] which showed an average reduction in HIV infection by 60% in circumcised compared to uncircumcised males. A recent study in Uganda revealed that although male circumcision lowered the HIV prevalence, it significantly increased high-risk sexual behaviour [48].

Our study employed methods of spatial analysis to evaluate the relations between spatial distributions and HIV prevalence among young people in Uganda. The importance of cluster analysis in epidemiology is the detection of aggregates of diseases as well as testing for the presence of available significant clusters by ascertaining whether diseases found in the same geographical location may be explained by chance or random occurrences [20,49]. This is because most risk and health-promoting behaviours are clustered in specific communities as opposed to whole communities [49]. The results of this study provide useful information on the existing epidemiological situation of HIV/AIDS in Uganda by highlighting the geographic differences of HIV infection in Uganda. Knowledge on the presence of HIV clusters at regional level can assist regional authorities in strengthening measures that effectively control the spread of HIV/AIDS among young people and mapping out future strategies [50]. Although HIV/AIDS in Uganda is generalized, this study revealed the Central and Eastern regions as being the most probable regions for new HIV infections. Health authorities should therefore investigate why these regions are more affected and identify the geographic factors propagating HIV/AIDS among young people.

### Strengths and limitations

The major strength of this study was use of spatial analytical techniques had advantages over standard statistical techniques to identify geographical variations of HIV prevalence in Uganda. This may be of public health significance in the fight against the spread of HIV/AIDS not only in Uganda but in other countries gravely affected by this scourge. Cluster analysis using the Scan Statistic method adjusts for population density and reduces selection bias as the clusters are explored without subsequent knowledge of their location, size or time period [51]. The use of the Bayesian approach, by adding a spatial random effect, reduces bias and inaccurate conclusions that would arise from ignoring spatial auto-correlation present in the associated factors [29]. As inherent with all cross-sectional studies, this study could neither establish temporality nor causality of the observed associations with the outcome. Self-reporting of sexual behaviours could have introduced recall or social desirability bias. Performing cluster analysis in areas of Uganda where the survey communities were further apart may possibly render the spatial scan statistic less robust. Finally, lack of geocodes in sampled areas failed to depict a correct representation of cluster analysis results with the Western most area of Uganda devoid of any spatial HIV clusters.

## Conclusions

The findings in this study indicated that marital status, age at sexual debut, STIs, alcohol use and condom use were important predictors of HIV infection among persons aged 15–24 years in Uganda. HIV prevention programmes in Uganda as well as in other developing neighbouring countries should focus on these factors in order to alleviate the spread of HIV/AIDS among young persons. HIV/AIDS prevention programmes aimed at young persons should include promoting the delay of coital debut which influences multiple sexual partnerships and leads to higher risk of HIV infection. Furthermore, emphasis on the risk of HIV infection after circumcision should be highlighted to reduce risky sexual behaviours among circumcised males.

Spatial analysis revealed the existence of clusters which may indicate presence of concentrated epidemics in an otherwise generalised epidemic. Identification of these clusters is crucial for targeted biomedical, behavioural and structural interventions that may reduce the burden of HIV/AIDS. The use of cluster detection techniques for surveillance of HIV/AIDS in different regions may help inform public health authorities in disease controlling activities. These findings indicate the need for policy makers to formulate more appropriate and region-specific management strategies in combating HIV/AIDS.

## Abbreviations

(U)AIS: (Uganda) AIDS Indicator Survey; AOR: Adjusted odds ratio; BCI: Bayesian credible intervals; (U)DHS: (Uganda) Demographic And Health Survey; EA: Enumeration areas; ESDA: Exploratory spatial data analytical; POR: Posterior odds ratio; SSA: Sub-Saharan Africa; STI: Sexually transmitted infection.

## Competing interests

The authors declare that they have no competing interests.

## Authors’ contributions

LAC undertook data request process from MEASURE DHS, mapping and data analysis. All authors contributed to the interpretation of the data. LAC wrote the first draft of the paper and EM read the draft and provided critical comments. Both authors read and approved the final draft of the paper.

## Pre-publication history

The pre-publication history for this paper can be accessed here:

http://www.biomedcentral.com/1471-2458/14/555/prepub
